# Escitalopram and Neuroendocrine Response in Healthy First-Degree Relatives to Depressed Patients – A Randomized Placebo-Controlled Trial

**DOI:** 10.1371/journal.pone.0021224

**Published:** 2011-06-27

**Authors:** Ulla Knorr, Maj Vinberg, Allan Hansen, Marianne Klose, Ulla Feldt-Rasmussen, Linda Hilsted, Jørgen Hasselstrøm, Ulrik Gether, Per Winkel, Christian Gluud, Jørn Wetterslev, Lars Vedel Kessing

**Affiliations:** 1 Department of Psychiatry Copenhagen, Rigshospitalet, Copenhagen University Hospital, Copenhagen, Denmark; 2 Department of Medical Endocrinology, Rigshospitalet, Copenhagen University Hospital, Copenhagen, Denmark; 3 Department of Clinical Biochemistry, Rigshospitalet, Copenhagen University Hospital, Copenhagen, Denmark; 4 Department of Clinical Biochemistry, Center for Psychiatric Research, Aarhus University Hospital, Aarhus, Denmark; 5 Center for Pharmacogenomics, Department of Neuroscience and Pharmacology, University of Copenhagen, Copenhagen, Denmark; 6 Copenhagen Trial Unit, Centre for Clinical Intervention Research, Rigshospitalet, Copenhagen University Hospital, Copenhagen, Denmark; Georgetown University Medical Center, United States of America

## Abstract

**Introduction:**

The mechanisms by which selective serotonin re-uptake inhibitors (SSRI) act in depressed patients remain unknown. The serotonergic neurotransmitter system and the hypothalamic-pituitary-adrenal (HPA) system may interact. The aim of the AGENDA trial was to investigate whether long-term intervention with SSRI versus placebo affects the cortisol response in the dexamethasone corticotropin-releasing hormone (DEX-CRH) test in healthy first-degree relatives to patients with major depressive disorder (MDD).

**Methods:**

Eighty healthy first-degree relatives to patients with MDD were randomized to escitalopram 10 mg versus matching placebo daily for four weeks. The primary outcome measure was the intervention difference in the change of the total area under the curve (CorAUC_total_) for plasma cortisol in the DEX-CRH test at entry to after four weeks of intervention.

**Results:**

Change in CorAUC_total_ showed no statistically significant difference between the escitalopram and the placebo group, *p* = 0.47. There were large intra- and inter-individual differences in the results of the DEX-CRH test. There was statistically significant negative correlation between the plasma escitalopram concentration and change in CorAUC_total_, rho = −0.41, *p* = 0.01. Post-hoc analyses showed a statistically significant interaction between age and intervention group and change in log CorAUC_total_.

**Conclusion:**

The present trial does not support an effect of escitalopram 10 mg daily compared with placebo on the HPA-axis in healthy first-degree relatives to patients with MDD. Increasing levels of escitalopram tended to decrease the HPA-response in the DEX-CRH test and this effect increased with age.

**Trial Registration:**

ClinicalTrials.gov [NCT00386841

## Introduction

Depression is associated with an altered function of the neuroendocrine feedback regulation of the hypothalamic-pituitary-adrenal (HPA) axis, including cortisol escape from dexamethasone suppression and increased cortisol responses to the dexamethasone corticotropin releasing hormone (DEX-CRH) test [1]. Previous studies have shown that even healthy first-degree relatives to patients with major depressive disorder (MDD) have an abnormal HPA response to the DEX-CRH test, with an intermediary response when compared to healthy controls and patients with major depression [2]. Furthermore, salivary cortisol has been shown to be increased in individuals with a family history of MDD as compared to healthy individuals without a family history of MDD [3]–[5]. Intervention with a single dose of a selective serotonin re-uptake inhibitor (SSRI) has been found to increase serum corticosterone levels in rats [6], [7] and plasma corticosteroid levels in healthy humans [8]–[12]. In rats plasma levels of HPA-axis hormones, corticosterone and adrenocorticotropic hormone (ACTH), decreased after 15 days intervention with citalopram [13]. It is well known that selective serotonin reuptake inhibitors (SSRI) have an effect on depression [14], [15]. Studies of depressed patients have suggested that improved hypothalamic-pituitary-adrenal (HPA) system regulation (decreased DEX-CRH test response) is associated with beneficial treatment response [16]. Whether this is a direct effect of treatment with antidepressants or a consequence of improvement in depressive symptoms is unclear.

Robins and Guze described five phases in the development of a valid classification of psychiatric illness: clinical description, laboratory studies, delimitation from other disorders, follow-up studies and family studies [17]. Later, response to treatment was added as a sixth phase [18]. Recently, the endophenotype concept has emerged as a strategic tool in neuropsychiatric research [19].

Endophenotypes are quantifiable components in the “genes-to-behaviours” pathways distinct from psychiatric symptoms [19]. In parallel with the classification of psychiatric diseases, endophenotypes are validated by specificity, state independence, heritability, familial association, co-segregation, and biological and clinically plausibility [20].

Several possible endophenotypes have been proposed in affective disorders, including stress regulation, cognition, neuroticism, depression and anxiety symptoms [20]. Pharmacological anti-depressants may have an effect on endophenotypes in healthy persons with a family history of depression. We hypothesized that treatment response could be added to the validation of possible endophenotypes for depression.

It is unclear whether antidepressants have an effect on potential endophenotypes for depression in healthy first-degree relatives of patients with depression, thus it has never been investigated whether this deregulated HPA axis in healthy individuals with a family history of MDD may become normalized by antidepressants [21]. The AGENDA (**A**ssociations between **g**enepolymorphisms, **e**ndophenotypes for **d**epression and **a**ntidepressant intervention) trial [22] is the first to investigate the effect of long-term (four weeks) daily administration of a selective serotonin re-uptake inhibitor (SSRI) versus placebo on the HPA-axis in healthy first-degree relatives to patients with MDD [21]. The function of the HPA-axis was investigated using the DEX-CRH test. The aim of the present trial was to test the hypothesis that an intervention with SSRI as compared with placebo decreases the cortisol response in the DEX-CRH test for first-degree relatives of patients with MDD.

The ultimate translational value of a experimental trial like the ADENDA trial of high-risk individuals is to increase our understanding of the patogenesis of illness.

## Materials and Methods

The AGENDA trial was investigator initiated and designed. The protocol for this trial and supporting CONSORT checklist are available as supporting information; see Checklist S1 and Protocol S1. It was conducted as a participant-, investigator-, observer-, and data-analyst-blinded trial. During the trial the participants received either escitalopram 10 mg/day or placebo for a period of four weeks. The trial protocol was published ahead of trial completion [22]. The trial was conducted from July 2007 until July 2009 at the Department of Psychiatry, Rigshospitalet, Denmark as part of the Centre for Pharmacogenomics, University of Copenhagen, Denmark. The trial was conducted and monitored in accordance with the International Conference on Harmonization for Good Clinical Practice guidelines and the Declaration of Helsinki 2002 (www.wma.net/e/policy/b3.htm). The Local Ethics Committee (De Videnskabsetiske Komitéer for Københavns og Frederiksberg Kommuner, Københavns Kommune) approved the trial: H-KF 307413.

### Probands

Probands were patients with MDD from psychiatric in- or out-patient hospital contact in Denmark who participated in ongoing studies at the Department of Psychiatry, Rigshospitalet. Their diagnoses were validated by face-to-face interviews including the semi-structured interview Schedules for Clinical Assessment in Neuropsychiatry (SCAN) [23] by trained medical doctors [24]. The probands were asked to permit that the first author contacted their adult children and siblings.

### Participants

Participants were recruited as healthy first-degree relatives (adult children or siblings) of the probands described above. Individuals meeting the inclusion criteria and none of the exclusion criteria were enrolled in the trial, Table 1.

**Table 1:** Criteria for inclusion and exclusion in the AGENDA trial.

Inclusion criteria	Exclusion criteria
Healthy individual of both sexes*	Somatic illness or other handicap, which made participation in the trial impossible
Offspring or sibling of an ethnic Dane, with a history of psychiatric in- or outpatient care with a diagnosis of major depressive disorder and who later had the diagnosis verified in a SCAN interview at the Department of Psychiatry Rigshospitalet, Denmark 2004–2009	Daily intake of drugs interfering with corticosteroids or escitalopram, including birth control pills or any kind of corticosteroids
Aged 18–60 years. Women were **preferably in lutheal phase menstrual cycle or post-menopausal at the time of randomization	Hypersensitivity to escitalopram, dexamethasone, or human corticotropin-releasing hormone
Born in Denmark	Former medical or psychological treatment for diseases in the affective or schizophrenic spectrum
European parents and grandparents	Current abuse of alcohol or psychotropic medication
Able and willing to sign informed consent	Pregnancy or breastfeeding

*A total of 6 participants with stable treated medical conditions were included: hypertensio arterialis (3), pancreatitis antea (1), hypothyroidism (1) and acne vulgaris (1).

**Women were in lutheal phase or postmenopausal at the times of assessments.

### Assessments

The first part of the assessment was a telephone interview of the potential participants. The individuals eligible were scheduled to meet at the clinic on two different days both before and following four weeks of intervention. On the first day the participants gave written informed consent after details of the trial were explained. Diagnoses were ascertained by the SCAN interview and the structured Clinical Interview for DSM-IV Axis II Personality Disorders [25]. The DEX-CRH test was performed at entry and following four weeks of intervention. Further assessment included information on family history of psychiatric disorders, ratings of mood using the 17-item Hamilton Depression Rating Scale (HAM-D) [26] and 14-item Hamilton Anxiety Scale [26], various socio-demographics, height, weight, routine blood tests, and, a pregnancy test for women. Furthermore, following four weeks of intervention blood was drawn for measurements of plasma escitalopram, and the UKU Side Effect Rating Scale [27] was applied by the principal investigator.

### Interventions

The participants were randomized to self-administer a single dose of either escitalopram 10 mg or placebo daily for four weeks. The manufacturer provided escitalopram and placebo tablets. The tablets were identical in appearance, color, smell, and solubility allowing for blinding of the assignment to intervention or placebo. An independent pharmacist packed the identically appearing blister packages containing escitalopram or placebo and then sealed, and numbered the packages according to a randomization list provided and concealed by the Copenhagen Trial Unit (CTU). On completion of the four weeks of intervention participants entered a five-day down-titration period to nil medication. Compliance to the protocol was sought by making weekly telephone calls to the enrolled participants. The participants were asked at the end of the trial, if they had missed taking any tablets.

### Randomization

CTU performed the centralized computerized randomization 1∶1 by telephone to secure adequate allocation sequence generation and allocation concealment. Randomization was stratified in blocks of 6, by age (18–31 and 32–60 years), and sex. Only the data manager knew the block size.

### Blinding

All trial personnel and participants were blinded to the packaging of the trial drug, and blinding was maintained throughout monitoring, follow-up, assessment of outcomes, data management, data analyses, and conclusions drawn [28]. At the assessment after four weeks intervention, each participant and the principal investigator (UK) made a guess as to which intervention the participant had received. The agreement between the actual intervention and the guesses was estimated to assess the degree to which blinding had been demasked, thus κ: <0 no; 0.0–0.20 = slight; 0.21–0.40 = some; 0.41–0.60 = moderate; 0.61–0.8 = substantial; 0.81–1.00 = almost complete demasking.

### Analysis of plasma escitalopram

The extraction and quantitation of escitalopram was carried out on an ASPECXL combined with a high-pressure liquid chromatography system, both from Gilson, Villiers le Bell, France.

Lower and upper limits of quantitation were 10 nmol/l and 3,600 nmol/l. The interassay coefficients of variation ranged from 5.5% to 8.4% and trueness ranged from 93.2% to 103.0% within the measurement range.

### Change in hormone responses to the combined DEX-CRH test

Cortisol and ACTH levels in response to the combined DEX-CRH test were measured before and after four weeks of intervention. The application of this combined DEX-CRH-challenge requires individuals to take 1.5 mg dexamethasone (DEX) at 23:00 h orally the previous night. On the day of the test, 100 micrograms human CRH are administered to the subjects under study at 15:00 h intravenously as a bolus, and blood samples for the determination of plasma cortisol and ACTH are drawn every 15 min from 14:00 h to 18:00 h. [2]. The participants had a light lunch at noon at the day of the CRH challenge. A trained bio-technician and trained medical students conducted the tests under the supervision of the principal investigator.

### Analyses of cortisol and ACTH

Hormones were analyzed at the Department of Clinical Biochemistry, Rigshospitalet. Plasma cortisol was measured using a competitive electro chemiluminescense immuno assay (ECLIA) (Roche Diagnostica Cortisol) and Modular analytics E170 (Roche). Lower and upper limits of quantiation were 1.0 and 17,500 nmol/l. The interassay coefficients of variation were 4.7% and 5.6% at 116 and 968 nmol/l, respectively. Plasma ACTH was measured using a sandwich chemiluminescense immunometric method (ACTH, Immulite Siemens DPC) and Siemens Immulite 2000. Lower and upper limits of quantitation were 1.0 and 556 pmol/l. The interassay coefficients of variation were 7.6% and 6.1% at 7 and 106 pmol/l, respectively.

In accordance with Modell *et al*, cortisol and ACTH responses were calculated according to the trapezoidal rule as the total area under the curve (AUC_total_) from administration of CRH at 15:00h to the last measure at 18:00h [2]. The plasma cortisol (COR) BASAL was estimated as the mean of the baseline measurements before the administration of CRH. CorPEAK was estimated as the highest plasma cortisol measurement following CRH administration. The primary outcome measure was the difference between the intervention groups change of the total area under the curve (CorAUC_total_) for plasma cortisol in the DEX-CRH test at entry to after four weeks of intervention, ΔCorAUC_total_. It was calculated by subtracting CorAUC_total_ at four weeks from the CorAUC_total_ determined immediately before the initiation of the intervention. Similarly, Δ was calculated for ACTH AUC_total_, CorBASAL, and CorPEAK. The ΔCorAUC_total_ was chosen since CorAUC_total_ was statistically significant in the Modell study.

### Statistical methods

The sample size estimation and the pre-established data analysis plan have previously been described [22]. The power calculations were hypothetical since the effect of SSRI on the DEX-CRH test in healthy has not been investigated in prior trials [22]. Thus, the power calculation was merely guided by a previous case control study in which the difference between healthy with and without a family history of MDD was regarded as a possible relevant difference [2], reflecting the hypothesis that the increased cortisol response to the DEX-CRH test in individuals with a family history of MDD would decrease, as a result of the SSRI intervention, to the level of the cortisol response measured in healthy without a family history of MDD (see below).

Data from all randomized participants were analyzed, including those with missing data on the DEX-CRH test. The primary outcome measure was not normally distributed, and could not be transformed into a normal distribution. Thus, the outcome in the intervention and the placebo groups were compared by the Mann-Whitney test.

Regression analyses (using the general linear univariate model) were done including the primary outcome as the dependent variable and a treatment indicator (escitalopram versus placebo) plus design variables, including stratification variables [29] age, sex, HAM-D total score at entry, body mass index at entry, number of daily cigarettes, and concentration of escitalopram in plasma as independent variables Exploratory analyses (including the primary outcome, the treatment indicator and one design variable) were first done to select the design variables (if any) to be included in the final analysis (if any). If the explorative analysis of a design variable had a p-value>0.1 the design variable was not included in the final analysis.

In these analyses the residuals followed a Gaussian distribution with reasonable approximation (the distributions were reasonably symmetric but most of them deviated significantly from a Gaussian one as judged from the Shapiro Wilks test).

Initially, the drug level measured in each participant was not included in the models as to keep the statistician and investigators blinded. Lastly, after all other analysis had been done and conclusions drawn, analyses for the effect of drug-level were performed.

An explorative analysis (not protocol specified) included the natural log of the ratio between the total cortisol AUC measured after intervention and the total cortisol AUC measured before the intervention (ln(deltaAUC)), the treatment indicator and the design variables. Ln(deltaAUC) followed the Gaussian distribution with good approximation. We first analysed if there was a treatment effect of this outcome measure in a regression analysis including ln(deltaAUC) and the treatment indicator (I). Then we examined if any of the design variables interacted with the treatment. Each analysis included ln(deltaAUC), the treatment indicator (I), one design variable (D) and I*D.

Explorative analyses were also done using a mixed model see e.g. Sullivan et al. [30] and Winkel et Zhang [31].

The modeling program (R-program) not including the intervention indicator was developed using the p-cortisol data measured at the start of the trial. The intervention indicator was then included (see below) in this program, which was applied on the p-cortisol values measured in the two intervention groups at the end of the trial.

Initial analyses showed that it was necessary to use logarithmic values to obtain homogeneity of variance. The p-cortisol was measured 17 times in each subject. Figure 2 shows the mean of ln(p-cortisol/unit) as a function of time in each of the two groups. It appears that the relationship between mean values and time (t) is linear with reasonable approximation within each of the intervals [1≤t≤5], [6≤t≤9] and [10≤t≤17] in the following referred to as A, B and C, respectively. The model is as follows:
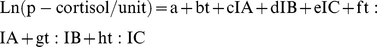
where a, b, c, d, e, f, g and h are coefficients (‘fixed effects’), to be estimated. IA is an indicator equal to 1, if t is within the time interval A and 0 otherwise. IB and IC are similarly defined; a is an intercept, b is the common slope of p-cortisol as a function of time. The indicators define the level of each of the three linear functions corresponding to the above-defined three time intervals. The interaction (symbolized by ‘:’) between t and the indicators serve to modify the slope of each of the three linear functions relative to the common slope b.

Significant reductions of the variability were obtained by supplementing the model with random effects (subject specific coefficients) corresponding to all of the above fixed effects.

An analysis of the variance of the residuals showed inhomogeneity of the variances in that the variance was not the same in the three groups defined by the three time intervals This effect was therefore included in the final model. The residuals were not significantly correlated.

Using the subject specific random effects in conjunction with the fixed effects personalized models of the p-cortisol versus time curve may be developed and used to follow a subject as his/her own control.

In the present context we utilized the model by introducing an intervention indicator (arm) in it (main effect and interactions, i.e. the terms arm, arm:t, arm:IA arm:IB etc were added to the model). We then tested if any of the corresponding coefficients differed significantly from 0 to see if the intervention influenced the three linear functions in any way.

In correlation analyses where the data did not follow Gaussian distributions we used the Friedman non-parametric test in place of the Pearson correlation test.

All analyses of the primary outcome were performed using cases without any missing values (complete case analysis), as well as using all cases completed by multiple imputation analysis of missing area under the curve of the DEX-CRH test (SAS version 9.1) [22]. The following quantities were included in the multiple imputations: age, sex, BMI-1, HAMD-1, HAMD-2, nScale-1, nScale-2, NEON-1, NEON-2, YearEducation, corAUCtotal-1, corAUCtotal-2, salivaryCorAUCtotal-1, salivaryCorAUCtotal-2, ActhAUCtotal-1, ActhAUCtotal-2, and Intervention. To normalize the quantities during the imputations the following transformations were done: age, BMI-1, and all areas were log transformed, HAMD-1 and HAMD-2, nScale-1, and nScale-2 were square root transformed. The transformations y1 = 1/NEON-1^2^ and y2 = 1/1/NEON-1^2^ were used to transform NEON-1 and NEON-2. Since the results obtained using complete case analysis and those obtained using analysis of 10 sets of imputed data were almost identical we only report the results of the complete case analysis.

## Results

### Participants and non-participants characteristics

**Figure 1:**
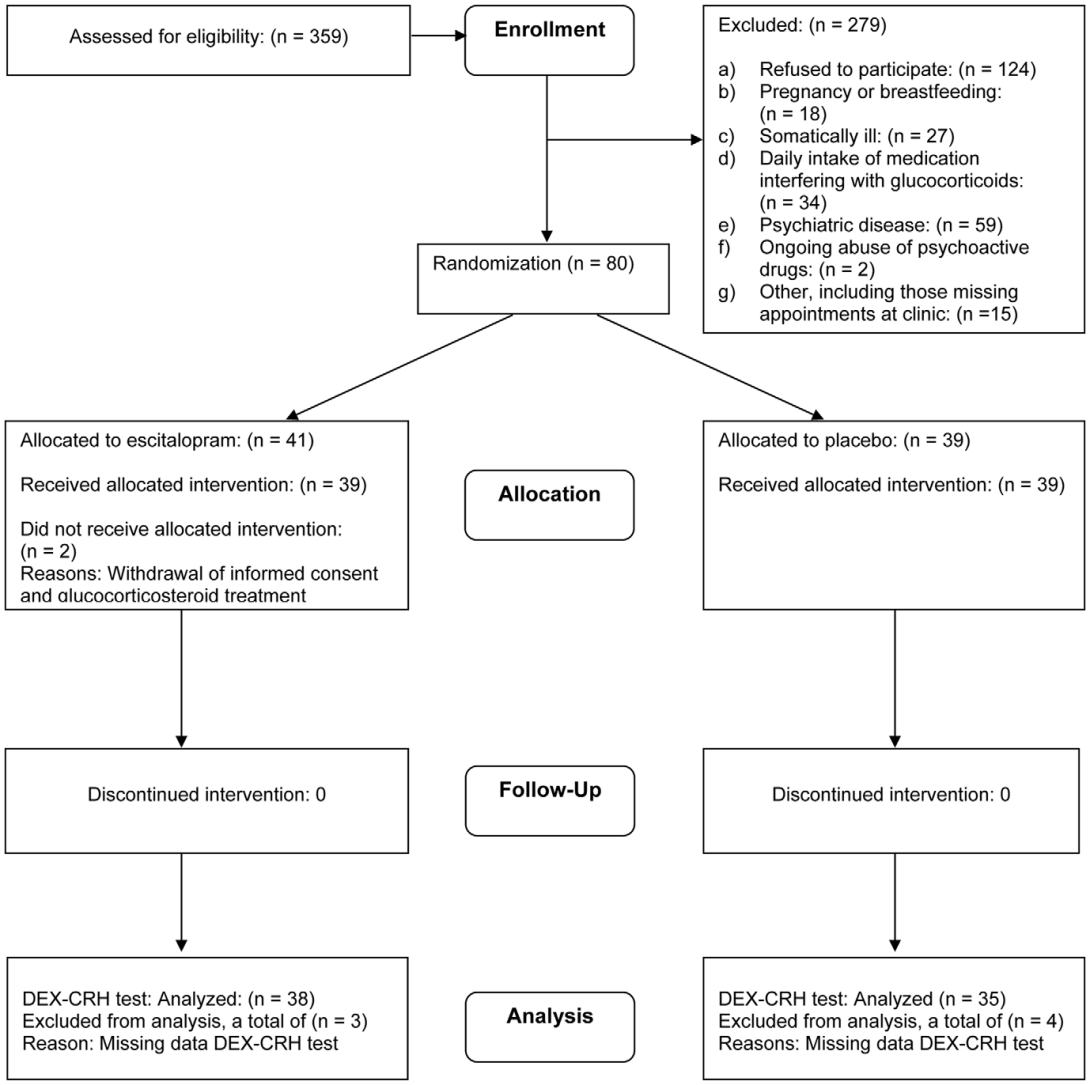
Flowchart for the AGENDA trial.

The probands (N = 466) gave us permission to contact 359 first-degree relatives, who were the potential participants in the trial. The CONSORT participant flow diagram is shown in Figure 1. A total of 80 participants, 29 (36%) women and 51 (64%) men were included and randomized. The clinical and demographic characteristics of the participants can be seen in Table 2.

**Table 2:** Characteristics of participants at entry.

Characteristic	Escitalopram (N = 41)	Placebo (N = 39)	All Participants (N = 80)
Age – yr, mean ± SD	32±11	31±11	32±10
Women – N (%)	15 (37)	14 (36)	29 (36)
Proband was / – N (%)			
sibling	18 (44)	15 (39)	33 (41)
parent	23 (56)	24 (62)	47 (59)
Caucasian – (%)	100	100	100
Education – mean ± SD			
Years of school	11±1	11±1	11±1
Years of further education	3±2	3±2	3±2
Employment status – N (%)			
Employed	30 (73)	26 (67)	56 (70)
Student	11 (27)	11 (28)	22 (28)
Unemployed	0 (0)	2 (5)	2 (3)
Marital status – N (%)			
Single	15 (37)	23 (59)	38 (48)
Married or cohabiting*	26 (63)	16 (41)	42 (52)
First degree relatives of patient with a history of major depressive disorder ……………..– median (quartiles)**	1 (1;2)	1 (1;2)	1 (1;2)
Second degree relatives with a history of major depressive disorder – median (quartiles)	0 (0;1)	0 (0;1)	0 (0;1)
17-item Hamilton Depression Scale Score, . – median (quartiles) (range)	1 (0;3) (0–7)	1 (0;3) (0–7)	1 (0;3) (0–7)
14-item Hamilton Anxiety Scale Score, – median (quartiles) (range)	1 (0;2)… (0–9)	1 (0;2) (0–6)	1 (0;2) (0–9)
Beck Depression Inventory, 21-item, depression median (quartiles)	2 (0;4)	2 (0;3)	2 (0;5)
Beck Depression Inventory, 14-item, anxiety – median (quartiles)	1 (0;4)	2 (0;3)	1 (0;3)
Body Mass Index – kg/m^2^, mean ± SD	25±4	26±5	26±4
Numbers of daily cigarettes - median (quartiles)	0 (0;11)	0 (0;10)	0 (0;10)
Package years – median (quartiles)	1 (0;10)	2 (0;7)	1.75 (0;8)
Daily medicine – N (%)	2 (5)	4 (10)	6 (8)
Plasma cortisol AUC_total_ - nmol/l×min/l, mean ± SD, median (quartiles)	9045±12829 4691 (2864;8277)	15126±17542 9974 (2549;18336)	12005±15506 5095 (2669;13833)
Plasma ACTH AUC_total_ - pmol/l×min/l, mean ± SD, median (quartiles)	324±272 255 (209;304)	365±197 306 (233;426)	343±239 263 (215;263)
Plasma cortisol BASAL - nmol/l, mean ± SD, median (quartiles)	15±15 13 (8;17)	24±37 15 (10;20)	19±28 14 (9;18)
Plasma cortisol PEAK - nmol/l, mean ± SD, median (quartiles)	90±124 41 (22;82)	137±153 86 (19;191)	112±140 52 (20;136)

Notes: Two smoked cannabis more than two months prior to the investigation. Three were previously abusing alcohol. One participant had generalized anxiety.

*Eight were living with their parents.

**quartiles reported, are the 25 and 75 quartiles.

There was no statistically significant difference (p>0.05) between the escitalopram and the placebo group for any of the hormone measures. AUC_total_ = Area under the curve after administration of CRH corrected for baseline equivalent, BASAL = mean of five measurements at the baseline after pre-treatment with deametasone 1.5 mg and before the administration of CRH, PEAK = the highest measurement following CRH administration.

The mean age of the non-participants was 37 (SD 11) years and 58% were women. The reasons for their non-participation are presented in Figure 1.

### The success of blinding

The agreement between the actual intervention group and the guess was ‘some’ demasking (κ = 0.23 (0.01–0.45)) for the participants and ‘slight’ demasking (κ = 0.18 (0.00–0.40)) for the principal investigator.

### Adherence to the intervention and adverse events

The validity of the results depended on a high compliance and high completion in the trial. This was sought obtained by weekly telephone control calls to the enrolled participants to insure adherence to the protocol and to record adverse events. Two participants randomized to escitalopram were excluded from the trial prior to intervention: one man withdrew informed consent, and one woman developed skin rash necessitating glucocorticosteroid treatment. No participants left the placebo group, and 33 in the escitalopram group and 32 in the placebo group stated full compliance with the protocol. Six participants in the escitalopram group and seven in the placebo group stated that they missed taking one or two tablets. No severe adverse reactions, or serious adverse events occurred. Following four weeks of intervention, 56% of the participants in the placebo group and 46% of the participants in the escitalopram group, reported no side effects. Adverse events are listed in Table 3.

**Table 3:** Assessed adverse events by the UKU Side Effect Rating Scale for 78 healthy first degree relatives of patients with a history of major depressive disorder following four weeks of intervention by escitalopram 10 mg (N = 39) or placebo (N = 39) in the AGENDA trial.

Adverse events	Escitalopram N (%)	Placebo N (%)	*p* (χ^2^)
Restlessness	6 (15)	9 (23)	0.39
Insomnia	2 (5)	9 (23)	0.02*
Tremor	1 (3)	1 (3)	1.00
Nausea	4 (10)	4 (10)	1.00
Diarrhoea	4 (10)	1 (3)	0.17
Sweating	6 (15)	4 (10)	0.50
Less libido	7 (18)	2 (5)	0.08
Erective dysfunction (men)	5 (13)	1 (3)	0.09
Ejaculating problems (men)	11 (28)	1 (3)	0.002*
Orgasmic dysfunction	11 (28)	0 (0)	0*
Headache	1 (3)	1 (3)	1.00

### Plasma escitalopram

Blood was drawn from all 78 participants at follow up, but one test from the escitalopram group failed. The mean concentration of escitalopram was 50 nmol/l, SD 29 nmol/l, median 48 nmol/l, range <10 to 138 nmol/l, (N = 38). Two participants from the escitalopram group had undetectable plasma escitalopram, thus <10 nmol/l, one of which had stated missing the last two tablets prior to blood sampling. Plasma escitalopram was undetectable in all participants of the placebo group.

### Cortisol and ACTH response in the DEX-CRH test

The two datasets for the DEX-CRH test were complete for 73 participants. Thus, two participants had no tests. Further, one woman and one male missed the baseline test due to schedule problems. The test following the intervention was missed by two males due to schedule problems and one male due to technical reasons.

There was no statistically significant difference of the primary outcome ΔCorAUC_total_ comparing the intervention and the placebo groups, (*p* = 0.47), (Table 4).

**Table 4:** The distributions of the primary outcome measure and other characteristics of plasma cortisol and plasma ACTH in the combined DEX-CRH test in 73 healthy first-degree relatives of patients with a history of major depressive disorder, in the escitalopram 10 mg group (N = 38) and the placebo group (N = 35).

Quantity	Group (N)	Mean (SD)	Median	Minimumvalue	Maximum value	Interquartile range	*p* b)
Δplasma cortisol AUC_total_ a)	Escitalopram	1675.1 (13001)	606.6	−40895.6	47913.8	8782.6	……0.47
	Placebo	1170.5 (17910)	−200.0	−44680.2	56859.7	7064.2	
Δplasma ACTH AUC_total_	Escitalopram	25.1 (158)	−0.08	−392.0	653.0	67.1	……0.23
	Placebo	−6.48 (255)	−10.7	−750.0	743.0	108.0	
Δplasma cortisol BASAL	Escitalopram	0.461 (13.5)	−0.345	−25.4	72.9	4.60	,,,,,,,0.57
	Placebo	5.17 (48.4)	0.340	−363	84.1	5.49	
Δplasma cortisol PEAK	Escitalopram	3.96 (124)	−3.92	−348	356	80.0	,,,,,,,0.61
	Placebo	1.76 (131)	1.23	−348	422	69.7	
ln (totalAUCafter/totalAUCbefore)	Escitalopram	0.039 (1.039)	0.147	−2.69	2.75	*−0.611 −0.292	,,,,,,,0.48
	Placebo	−0.120 (0.895)	−0.0265	−3.40	1.11	*−0.614 −0.294	

Δ was the difference between the measurement of plasma cortisol and ACTH after and before four weeks of intervention with escitalopram 10 mg or placebo for: AUC_total_ = Area under the curve after administration of CRH corrected for baseline equivalent, BASAL = mean of five measurements at the baseline after pre-treatment with dexametasone 1.5 mg and before the administration of CRH, PEAK = the highest measurement following CRH administration,

a) Δplasma cortisol AUC_total_ was the primary outcome measure.

b) *p* of Mann-Whitney test comparing the two distributions which did not follow normal distributions (Shapiro Wilkes test).

*95% C.I. are reported since the distributions followed normal distributions.

In univariate analyses, no statistically significant correlations were found between ΔCorAUC_total_ and the variables: age, sex, HAM-D, body mass index, and number of daily cigarettes at randomization (results not presented). We found no significant differences between the results of the complete-case analysis and the analysis done after multiple imputations (results not presented).

The correlation between plasma escitalopram and ΔCorAUC_total_ were analyzed in the escitalopram group. Increasing plasma escitalopram was significantly correlated with decreasing ΔCorAUC_total_, (Friedmanns rho = −0.41 (R^2^ = 0.046), *p* = 0.01).

### Post-hoc explorative analyses

The escitalopram group and the placebo group did not separate significantly in analyses of Δplasma ACTH AUC_total_, ΔCorBASAL, or ΔCorPEAK, Table 4. In additional analyses we found that the logarithm of AUC_total_ for plasma cortisol before and after the intervention followed a normal distribution with good approximation. Thus, the measure: ΔlogCorAUC = ln(CorAUC_total.after_)−ln(CorAUC_total.before_) = ln(CorAUC_total.after_/CorAUC_total.before_) = ln(ratio), which has a normal distribution, was analyzed. The means of ΔlogCorAUC for escitalopram versus placebo did, however, not differ significantly, (*p* = 0.49), Table 4.

There was a statistically significant interaction for ΔlogCorAUC between age and intervention group. Thus, the slope relating to age ΔlogCorAUC, (*p* = 0.024) differed significantly between the two intervention groups and the correlations between age and ΔlogCorAUC were R^2^ = 0.07, (Pearson's rho = −0.27), for escitalopram and R^2^ = 0.08, (Pearsons's rho = 0.28) for placebo, thus the escitalopram was associated with decrease in the HPA-response in the DEX-CRH test and this association increased with age.

**Figure 2:**
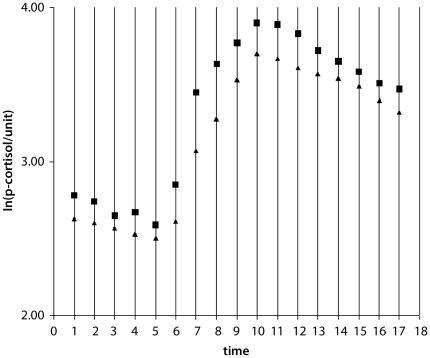
The mean of ln (p-cortisol/unit) versus time in the placebo group (squares) and in the escitalopram group (triangles). For each of the time intervals [1≤time≤5], [6≤time≤9] and [10≤time≤17] there is an approximate linear relationship between time and mean values.

Data were moreover analyzed using mixed model effect analyses. No statistically significant main effect of the intervention (*p* = 0.37) was noted and the intervention did nor influence the locations (*p* = 0.54) nor the slopes (*p* = 0.98) of the three linear functions (see Figure 2) significantly. In accordance with Modell *et al.* [2], a subgroup of 23 individuals with a PEAK cortisol concentration of 110 nmol/l or more in the DEX-CRH test at trial entry was analyzed. No statistically significant difference was shown on the ΔCorAUC_total_ for this subgroup, (*p* = 0.9). In addition, we analyzed the effect of escitalopram on ΔCorAUC_total_ for participants of the escitalopram group that had detectable escitalopram in plasma (N = 36) versus placebo, but no statistically significant difference was found, (*p* = 0.69).

## Discussion

The AGENDA trial is the first trial in which the effect of SSRI in healthy first-degree relatives of patients with depression has been investigated. Additionally, the AGENDA trial is the largest trial hitherto (N = 80) in which the effect of SSRI is investigated in healthy individuals regardless of outcome [21]. The main finding was that four weeks of intervention with escitalopram 10 mg/day compared with placebo had no statistically significant effect on neuroendocrine responses in the HPA-axis, as measured by ΔCorAUC_total_ in the DEX-CRH test, in healthy first-degree relatives of patients with MDD. Thus, our hypothesis that an intervention with escitalopram 10 mg would decrease the cortisol response in DEX-CRH test in healthy first-degree relatives of patients with MDD was not supported. Further, no statistically significant effect was found on any other measure of the DEX-CRH test (Table 4). Post-hoc analyses showed that increasing levels of escitalopram tended to decrease the HPA-response in the DEX-CRH test and this effect increased with age.

### Advantages of the trial

The AGENDA trial has several advantages. Firstly, the trial and the analyses were carried out as planned in advance and the completion and compliance in the trial was very high. Secondly, the registered diagnosis of depression for the probands was verified by a face-to-face psychiatric research interview by trained medical doctors. The participants were assessed and diagnosed by validated and frequently used multi-dimensional methods. Thirdly, the participants were genetically homogeneous as all were ethnic Danes with European, mostly Danish, parents and grandparents. Fourthly, we used well established methods, e.g., the DEX-CRH test which is a sensitive, biological, objective test to detect increased HPA-function in humans [1], [32]. The response to the DEX-CRH test may be sensitive to age [32] and sex [33], and in our trial, stratification by these factors resulted in equal distributions in the two intervention groups. Fifthly, the participants were studied in a randomized clinical trial blinded in all phases including the statistical analyses and conclusion phase. The blinding was successful in relation to participants as well as researchers. Finally, the antidepressant effect of escitalopram is generally accepted [14] and the participants were subjected to four weeks of intervention thus including the interval in which clinical improvement has been reported in trials with patients with MDD [14].

### Limitations

We have not compared healthy individuals with a family history of MDD to healthy individuals without a family history of MDD. However, the participants included in the present trial presented with values of CorAUC_total_ in the initial DEX-CRH test before intervention, (Table 2 (12,005±15,506 nmol/l×min/l)) that were higher than values found among healthy individuals without a family history of MDD in the study by Modell *et al.* [2] (7,773±1,071 nmol/l×min/l) and approaching the values for healthy individuals with a family history of MDD in that study (15064±3947 nmol/l×min/l), suggesting that participants included in our trial were comparable to the participants with a family history of depression in the Modell study. Notably, our results showed rather large intra- and interindividual variation in the baseline cortisol response to the DEX-CRH test as well in the change in the cortisol response from start to end of intervention, Tables 2 and 4. It cannot be excluded that the quality of the detective methods in the DEX-CRH test as well as the heterogeneity of the individuals included in the trial may have added to the increase in the variability of the DEX-CRH response. For example, the experimental situation of the DEX-CRH test may influence individuals in different ways resulting in a variation of the DEX-CRH response. However, this was a randomized trial and such unknown confounders ought to be evenly distributed between the escitalopram and the placebo group.

We cannot exclude that the dosage of 10 mg escitalopram was too low. However, this dosage has been suggested as the optimum dose for treatment of moderate depression [34] and it resulted in well-known adverse effects (Table 3), thus a substantial proportion of the participants in the escitalopram group reported sexual adverse effects. Notably, insomnia was decreased in the escitalopram group.

### Risk of errors

The risk of errors in trials falls in three major categories [35], [36]: 1) *Systematic error (‘bias’)*: We have minimized bias by using a randomized, age-and sex-stratified, comparison with blinding in all phases of the trial. 2) *Random error (‘play of chance’)*: We planned to include 80 participants due to resources, feasibility, and availability of the healthy first-degree relatives of patients with MDD studied in our group. Since no prior trials have investigated the effect of SSRI on healthy individuals, the power calculations were hypothetical and influenced by great uncertainty. Thus, we cannot exclude the possibility of overlooking a difference due to the play of chance. However, in the era of systematic reviews it has been questioned if the size of an individual trial still does matter [35]. The results from any trial may contribute to the larger body of evidence despite arbitrary sample size calculations in the individual trial that may eventually prevent important trials from being conducted [37]. 3) *Design errors*: These errors may include that some participants may not have reached sufficient levels of escitalopram in the blood in order to produce an effect on the HPA-axis. Our serum escitalopram concentrations were lower than in a study by Soegaard *et al.* [38], who found steady state plasma escitalopram concentrations of 63±32 nmol/l for escitalopram 10 mg as compared to 50±29 nmol/l in our trial. The low plasma levels in our trial may be a result of the fact that approximately 12 hours elapsed from taking the last tablet to blood sampling and that the half-life of escitalopram is 27–32 hours. However, a therapeutic plasma interval level has never been clearly defined for escitalopram. The intervention time is considered to be appropriate since clinical improvement is seen within this time, however it cannot be excluded that an effect of escitalopram could be detected in a trial with longer duration.

### Generalizability, clinical implication and future studies

Our participants were healthy, ethnic Danes, with a parent or a sibling who was treated for depression in a hospital setting in Denmark. Our results may generalize to healthy Caucasians in general. To infer direct clinical implications from the results were not an aim of our trial, but effects by escitalopram 10 mg on the primary outcome for the HPA-axis function in healthy was not detected. Future studies may explore individuals in prodromal phases of depressive disorder or establish a run in period to optimize adherence to protocols. Further, the distinction between healthy participants with and without increased familial risk for MDD needs further exploration.

### Interpretation

Considering advantages, disadvantages, risk of errors, and generalizability of the findings in this trial, it is likely that the results reflect reality. Thus, activation of the monoaminergic neurotransmitter systems by escitalopram does not seem to substantially affect the HPA-axis as measured by the DEX-CRH test in healthy individuals with a family history of depression. This finding seems to indicate that intervention with SSRI does not reduce the response to stress, as induced by CRH in the DEX-CRH test, in first-degree relatives. Our finding is in accordance with recent data showing that restoration of HPA system dysfunction seems to be neither a necessary nor a sufficient determinant for an acute treatment response in depressed patients [39]. Taken together these findings suggest that dysregulation of the HPA-axis, as assessed by the DEX-CRH test, does not play a *primary* role in the mechanisms of action of SSRIs. The HPA dysregulation seen in depressed patients may rather represent the down stream effects of other, more primary abnormalities as suggested by Manji *et al.* [40].

In conclusion, the AGENDA trial is the first to investigate the effect of a long-term intervention with escitalopram on serotonin-mediated HPA-axis responses in healthy first-degree relatives of patients with MDD. The results did not show a statistically significant difference in ΔCorAUC_total_ in the DEX-CRH test between escitalopram 10 mg and placebo given for four weeks. Further, the results showed large intra- and inter-individual differences in the response to the DEX-CRH test. Increasing drug levels of escitalopram was associated with a decrease in the HPA-response of the DEX-CRH test and this association increased with age.
